# Febuxostat effectively reduces uric acid but has a limited renoprotective effect on renal transplant recipients with hyperuricemia: a meta-analysis

**DOI:** 10.3389/fphar.2026.1728485

**Published:** 2026-02-25

**Authors:** Sheng Chao, Kejing Zhu, Lei Jia, Yulin Liu

**Affiliations:** Organ Transplantation Department, The Affiliated Hospital of Guizhou Medical University, Guiyang, Guizhou, China

**Keywords:** febuxostat, hyperuricemia, meta-analysis, renal transplant, uric acid

## Abstract

**Objective:**

Several previous studies have indicated that febuxostat can reduce uric acid (UA) levels and has a renoprotective effect on renal transplant recipients with hyperuricemia, but a comprehensive analysis of this effect is lacking. This meta-analysis aimed to analyze the effects of febuxostat on UA and renal function in renal transplant recipients with hyperuricemic disease.

**Methods:**

Web of Science, PubMed, the Cochrane Library, Wan Fang, and CNKI were searched up to 17 October 2024.

**Results:**

In renal transplant recipients with hyperuricemia, febuxostat decreased the UA level, with an MD of 129.981 μmol/L (*P* < 0.001). Creatinine (Cr) decreased (*P* = 0.337), whereas the estimated glomerular filtration rate (eGFR) increased, with a mean difference of −1.878 mL/min/1.73 m^2^, reaching a margin of statistical significance (*P* = 0.075) after the administration of febuxostat. In terms of other biochemical indices, febuxostat increased only hemoglobin (*P* = 0.008) but did not affect white blood cells, aspartate transaminase, or alanine aminotransferase (all *P* > 0.05). Sensitivity analysis revealed that the omission of most studies did not affect the study findings. The quality of the included studies was acceptable, and no publication bias existed.

**Conclusion:**

Febuxostat has a satisfactory UA-lowering effect, but its renoprotective effect is uncertain in renal transplant recipients with hyperuricemia. More studies are warranted to further explore its role in improving the prognosis of these patients.

**Systematic Review Registration:**

https://www.crd.york.ac.uk/PROSPERO/view/CRD420261300034.

## Introduction

Hyperuricemia is considered one of the most common complications in patients receiving renal transplantation because of the inability to excrete uric acid in time, which is derived from impaired renal function, the intake of immunosuppression drugs, and immunologic factors (Zhang X. et al., 2022; Cheng et al., 2020; Numakura et al., 2012; Einollahi et al., 2011; Kalantar et al., 2011). The incidence of hyperuricemia ranges from 19% to 84% in renal transplant recipients (Clive, 2000). In addition, hyperuricemia also results in a considerable disease burden for patients receiving renal transplantation (Nakagawa et al., 2006; Zi et al., 2022; Zhang F. et al., 2022; Hu et al., 2021). In detail, the occurrence of hyperuricemia in renal transplant recipients could further impair their renal function via the deposition of uric acid (UA) crystals in the transplanted kidney, disturbing the renin‒angiotensin system, causing renal hypertension, and damaging endothelial cell function (Nakagawa et al., 2006). Hence, timely and effective reduction of UA in renal transplant recipients with hyperuricemia is essential.

Febuxostat, a xanthine oxidase inhibitor, can reduce the level of UA by inhibiting the synthesis of UA (National Library of Medicine, 2006; Quintana et al., 2023). The UA-lowering effect of febuxostat has been preliminarily explored in several studies in renal transplant recipients with hyperuricemia (Li et al., 2019; Shen et al., 2019). For example, one study indicated that febuxostat might reduce the UA level from 470.82 ± 34.37 μmol/L to 378.77 ± 51.97 μmol/L and increase the estimated glomerular filtration rate (eGFR) from 75.55 mL/min/1.73 m^2^ to 85.23 mL/min/1.73 m^2^ (Li et al., 2019). In another study, after febuxostat was administered for renal transplantation combined with hyperuricemia, 62.5% of patients reached the target UA level, and the eGFRs also gradually increased (Shen et al., 2019). Even though some META analysis have explored the efficacy of febuxostat in other population such as population with hyperuricemic patients with or without gout and chronic kidney disease stage 3–5 patients with asymptomatic hyperuricemia (Chen et al., 2025; Fan et al., 2020). However, there is still a lack of conclusive evidence concerning the efficacy and safety of febuxostat in patients with renal transplantation combined with hyperuricemia.

Hence, this meta-analysis aimed to comprehensively analyze the UA-lowering and renal-protective effects and safety profile of febuxostat in patients with renal transplantation combined with hyperuricemia.

## Methods

### Search scheme and eligibility criteria

The literature retrieval databases, including Web of Science, PubMed, the Cochrane Library, Wan Fang, and CNKI, were searched up to 17 October 2024. The following keywords were used to search: ‘febuxostat’, ‘uloric’, ‘TEI-6720’, ‘hyperuricemia’, ‘HUA’, ‘kidney transplant’, ‘renal transplant’, ‘kidney transplantation’, ‘renal transplantation’, ‘kidney grafting’, and ‘renal grafting’. Searches were carried out in each database according to their unique retrieval mode. The search strategies for PubMed and Cochrane were listed in the Supplementary Table S1.

Studies were included if they 1) reported that patients had hyperuricemia post kidney transplantation; 2) reported that patients were aged >18 years; 3) reported that patients received febuxostat for therapy; and 4) reported data about the efficacy of febuxostat in the treatment of hyperuricemia post kidney transplantation and/or other laboratory indices (both pretreatment and posttreatment). Studies were excluded if 1) they were duplications, reviews or meta-analyses, case reports, or animal research; 2) they lacked data extraction for meta-analysis; or 3) there was an overlap in the data set between studies. This study was registered on the PROSPERO with the registration number of CRD420261300034.

### Quality assessment

The cohort studies were assessed by the Newcastle‒Ottawa Scale, with total scores ranging from 0 to 9. Higher scores indicated better quality (Wells et al., 2014). The Newcastle-Ottawa Scale evaluates risk of bias across three domains, including selection of participants, comparability of cohorts (reflecting adjustment for confounding), and outcome assessment. Single-arm studies were assessed by the methodological index for nonrandomized studies tool, which addressed methodological bias related to patient selection, outcome measurement, and follow-up. The first 8 items were used, each with a score of 0–2. The total score is 16, with higher scores indicating better quality (Slim et al., 2003).

### Data extraction

The first author’s name, publication year, country, sample size, mean age, male percentage, mean body mass index (BMI), febuxostat dose, and months post-treatment were extracted. Efficacy-related data on febuxostat in the treatment of hyperuricemia post kidney transplantation and/or other laboratory indices were screened. When more than or equal to 3 studies reported an index simultaneously, the index was included in the final analysis. In this meta-analysis, UA, creatinine (Cr), and eGFR were analyzed.

### Statistical analysis

The meta-analysis was performed with R ver.4.3.3. The mean difference (MD) with a 95% confidence interval (CI) was used to synthesize the pooled effects. The difference between pretreatment and posttreatment values was defined as the value before treatment minus the value after treatment. A random or fixed effect model was used to determine whether heterogeneity existed (*I* ^2^ > 50.0%). Begg’s or Egger’s test was used to analyze publication bias, and a funnel plot was drawn. Studies were omitted one by one to evaluate the reliability and robustness of the models for sensitivity analysis. Subgroups analyses were used to compare subgroup differences. Univariate meta-regression analyses were conducted to explore potential sources of heterogeneity. A *P* value less than 0.05 indicates statistical significance.

## Results

### Study flowchart and features of the included studies

**FIGURE 1:**
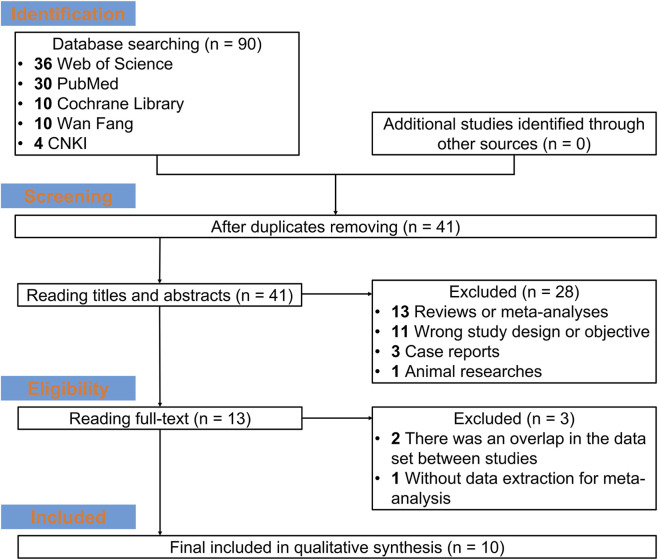
Study flow.

Studies were extracted from Web of Science, PubMed, the Cochrane Library, Wan Fang, and CNKI. A total of 90 studies were found, including 36 studies from the Web of Science database, 30 from the PubMed database, ten from the Cochrane Library database, 10 from the Wan Fang database, and four from the CNKI database. After the duplicate studies were removed, 41 studies were retained for reading the title and abstract. Then, 28 studies were excluded because they were reviews or meta-analyses (n = 13), had incorrect study designs or objectives (n = 11), were case reports (n = 3), or were animal studies (n = 1). The full texts of the remaining studies were subsequently read, and ten studies were ultimately included in the meta-analysis (Figure 1). Among the ten included studies, two were from Japan, one was from Korea, one was from Spain, and six were from China (Li et al., 2019; Shen et al., 2019; Sofue et al., 2014; Tojimbara et al., 2014; Baek et al., 2018; Ferreira et al., 2018; Jiang et al., 2019; Zhu et al., 2019; Liu et al., 2020; Xu and Zeng, 2021). A total of 557 patients were included, and the primary outcomes included UA, Cr, eGFR, WBC, Hb, ALT, and AST. Detailed information on the studies is shown in Table 1 and Supplementary Table S2.

**TABLE 1:** Features of included studies.

Study ID	Country	Sample size	Mean age (year)	Male (n)	Mean BMI (kg/m^2^)	Dose of febuxostat (mg/d)	Months of posttreatment (months)	Outcomes
Sofue et al. (2014)	Japan	15	52.0	13	22.2	20	3	UA
Tojimbara et al. (2014)	Japan	22	56.0	NR	NR	10–20	3	UA, Cr, eGFR, WBC, Hb, ALT, AST
Baek et al. (2018)	Korea	13	42.5	11	23.5	40–80	3	UA, eGFR
Ferreira et al. (2018)	Spain	15	55.6	14	NR	40	6	UA, Cr, eGFR
Shen et al. (2019)	China	48	35.5	12	21.1	40	3	UA, WBC, Hb, ALT, AST
Jiang et al. (2019)	China	124	39.0	89	20.3	10–40	3	UA, Cr, eGFR, WBC, Hb
Li et al. (2019)	China	22	43.8	20	21.9	20	3	UA, eGFR, Hb, ALT, AST
Zhu et al. (2019)	China	104	47.5	63	NR	40	3	UA, Cr, WBC, ALT
Liu et al. (2020)	China	164	37.0	90	21.5	20–40	3	UA, Cr, eGFR, WBC, Hb
Xu and Zeng (2021)	China	30	43.6	19	NR	40	3	UA

BMI, body mass index; UA, uric acid; NR, not reported; Cr, creatinine; eGFR, estimated glomerular filtration rate; WBC, white blood cell; Hb, hemoglobin; ALT, aspartate transaminase; AST, alanine aminotransferase.

Special statement: ‘Outcomes’ were defined as indices reported synchronously (among more than or equal to 3 studies) that could be used for meta-analysis.

### Effect of febuxostat on UA

**FIGURE 2:**
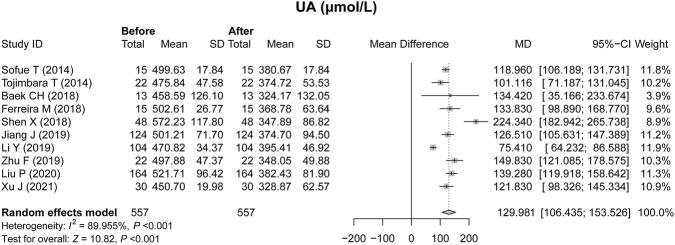
Effect of febuxostat on the UA level in renal transplant recipients with hyperuricemia.

All ten studies assessed the effect of febuxostat on UA in renal transplant recipients with hyperuricemia. Heterogeneity existed among the studies, and the random effects model was applied (*P* < 0.001, Figure 2). UA decreased after treatment with febuxostat, with an MD of 129.981 μmol/L (*P* < 0.001, Figure 2).

### Effect of febuxostat on renal function

**FIGURE 3:**
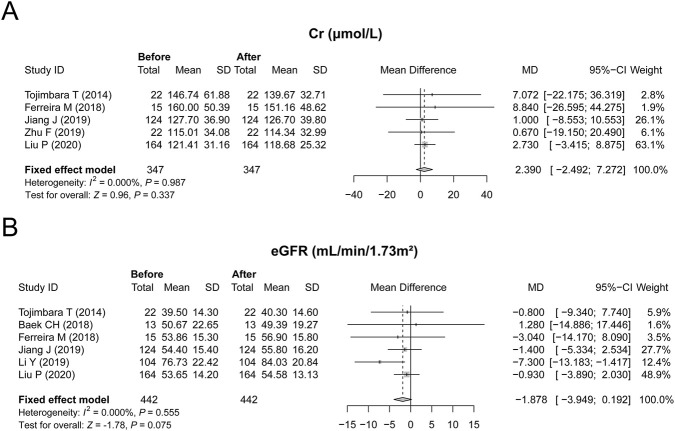
Effect of febuxostat on renal function in renal transplant recipients with hyperuricemia. Febuxostat did not affect the Cr level **(A)** and had a limited effect on the eGFR **(B)**.

Furthermore, renal function-related indices, including Cr and the eGFR, were also analyzed. Specifically, five studies reported changes in Cr without heterogeneity among these studies (*P* = 0.987, Figure 3A); hence, a fixed effects model was applied. Febuxostat numerically reduced the Cr level but did not reach statistical significance, with an MD of 2.390 μmol/L (*P* = 0.337, Figure 3A). Similarly, six studies reported the effect of febuxostat on the eGFR; no heterogeneity was observed among these studies, and a fixed effect model was applied (*P* = 0.555, Figure 3B). Febuxostat elevated the eGFR with an MD of −1.878 mL/min/1.73 m^2^, reaching a margin of statistical significance (*P* = 0.075, Figure 3B). These findings indicated that febuxostat only had a renoprotective effect to some extent, but this effect was limited.

### Effects of febuxostat on other biochemical indices

**FIGURE 4:**
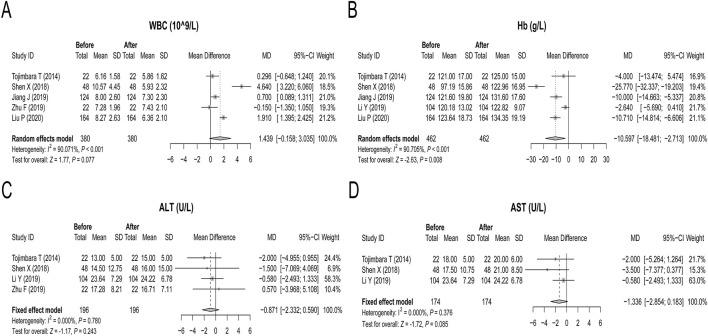
Effects of febuxostat on other biochemical indices in renal transplant recipients with hyperuricemia. Febuxostat had a limited effect on the WBC **(A)**. Febuxostat increased the Hb level **(B)**. Febuxostat had a limited effect on ALT **(C)** and AST **(D)** levels.

Five studies reported changes in WBC counts after febuxostat treatment, and a random effects model was applied because of heterogeneity (*P* < 0.001, Figure 4A). Febuxostat decreased the WBC count, and the statistical power reached the boundary of significance (*P* = 0.077, Figure 4A). Five studies described the effect of febuxostat treatment on Hb levels. Heterogeneity existed among these five studies; hence, the random effects model was applied. After treatment with febuxostat, the Hb concentration increased, with an MD of −10.597 g/L (*P* = 0.008, Figure 4B). Four and 3 studies reported ALT and AST levels, respectively, and fixed effects models were applied because there was no heterogeneity (both *P* > 0.05, Figures 4C,D). Febuxostat did not affect ALT or AST (both *P* > 0.05, Figures 4C,D). After reviewing the included studies on adverse events, only one study reported the rare adverse events, which reported that febuxostat induced an abnormal liver function (Baek et al., 2018).

### Sensitivity analysis

**FIGURE 5:**
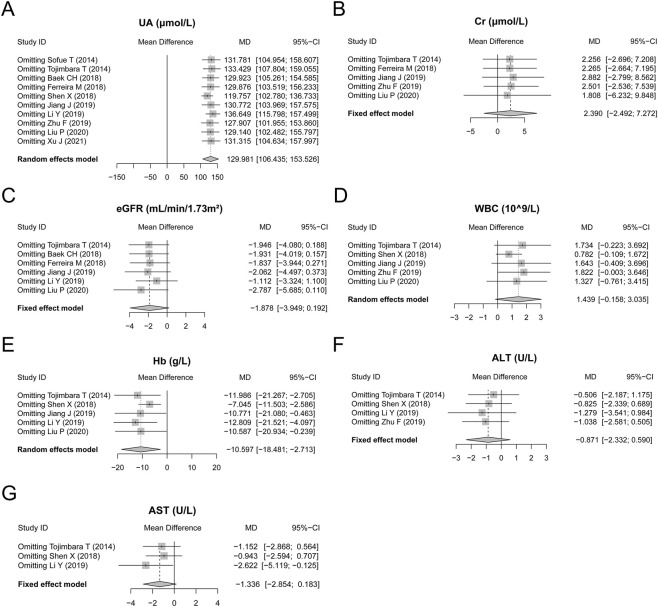
Sensitivity analysis. Sensitivity analysis of UA **(A)**, Cr **(B)**, eGFR **(C)**, WBC **(D)**, Hb **(E)**, ALT **(F)**, and AST **(G)** levels.

After sensitivity analysis, almost all the results, including the UA, Cr, eGFR, WBC, Hb, ALT, and AST levels and their corresponding significance, did not change after omitting any of these studies, except that the AST findings became statistically significant after omitting the study of Li et al. (2019). These findings indicated that the robustness of the results was acceptable in this meta-analysis (Figures 5A–G).

### Subgroup analysis

The detailed levels of UA, Cr, eGFR, WBC, Hb, ALT, and AST were shown in Supplementary Table S3. The subgroup analysis was carried out based on the study design (cohort study vs. single-arm study), dose of febuxostat (<40 mg/d vs. ≥40 mg/d), and follow-up duration (>6 months vs. ≤6 months). It indicated that both study designs showed a significant reduction in the UA level (both *P* < 0.001). The UA level was not different between the cohort studies and single-arm studies by random effect model (*P* = 0.664), while it was lower in cohort studies compared with the single-arm studies by common effect model (*P* < 0.001, Supplementary Table S4). Regarding the subgroup analysis based on dose of febuxostat (<40 mg/d vs. ≥40 mg/d), it indicated that both the <40 mg/d and ≥40 mg/d subgroups showed a significant reduction of UA level, while this trend seemed to be more obvious in subgroup of ≥40 mg/d without statistical significance (*P* = 0.067, Supplementary Table S5). Regarding the subgroup analysis based on follow-up duration, it indicated that both >6 months and ≤6 months subgroups had a significant reduction in UA level, while there was no subgroup difference (*P* = 0.490, Supplementary Table S5).

### Meta-regression analyses

The univariable meta-regression analyses were performed based on the UA, WBC, and Hb level. Regarding the UA level, the study type (*P* = 0.694), country (*P* = 0.509), and treatment duration (*P* = 0.951) were not associated with the UA level, while higher dose of febuxostat was related to the lower UA level (*P* = 0.019, Supplementary Table S6). In terms of the WBC level, the study type (*P* = 0.373), country (*P* = 0.523), and dose of febuxostat (*P* = 0.372) were not correlated with WBC level, while baseline UV level (per μmol/L) was associated with lower WBC level (*P* < 0.001, Supplementary Table S6). In regards to the Hb level, study type (*P* = 0.962) and country (*P* = 0.465) were not related to Hb level; however, dose of febuxostat (*P* < 0.001) and baseline UV level (*P* < 0.001) were associated higher Hb level (Supplementary Table S6). Furthermore, the baseline UA level (484.17 ± 20.84 vs.502.43 ± 42.29 μmol/L, *P* = 0.452) and UA achievement rate (61.5% vs. 73.5%, *P* = 0.327) were not different between non-China and China subgroups.

### Quality assessment

According to the Newcastle‒Ottawa scale, quality assessment was carried out on the cohort studies. The findings indicated that the quality of all 6 cohort studies included was high, and the overall scores ranged from 8 to 9 (Table 2). According to the scale of the methodological index for nonrandomized studies, the quality of the nonrandomized studies was high, including the item of a clearly state aim, item of inclusion of consecutive patients, item of prospective collection of data, item of endpoints appropriate to the aim of the study, item of unbiased assessment of the study endpoint, item of follow-up period appropriate to the aim of the study, item of loss to follow-up less than 5%, and item of prospective calculation of the study size (Table 3).

**TABLE 2:** Quality assessment of cohort studies via the NOS.

Study ID	Domain 1	Domain 2	Domain 3	Overall
Sofue et al. (2014)	4	2	3	9
Tojimbara et al. (2014)	4	2	2	8
Baek et al. (2018)	4	2	3	9
Shen et al. (2019)	4	2	2	8
Li et al. (2019)	4	2	2	8
Xu and Zeng (2021)	4	2	2	8

NOS, Newcastle‒Ottawa Scale.

Contents: Domain 1. Selection; Domain 2. Comparison; Domain 3. Outcome.

**TABLE 3:** Quality assessment of single-arm studies via MINORS.

Study ID	Item 1	Item 2	Item 3	Item 4	Item 5	Item 6	Item 7	Item 8
Ferreira et al. (2018)	2	2	2	1	0	2	2	0
Jiang et al. (2019)	2	2	2	2	0	2	2	0
Zhu et al. (2019)	2	2	2	2	0	2	2	0
Liu et al. (2020)	2	2	2	1	0	2	2	0

MINORS, methodological index for nonrandomized studies.

Contents: Item 1. A clearly state aim; Item 2. Inclusion of consecutive patients; Item 3. Prospective collection of data; Item 4. Endpoints appropriate to the aim of the study; Item 5. Unbiased assessment of the study endpoint; Item 6. Follow-up period appropriate for the aim of the study; Item 7. Loss to follow-up less than 5%; Item 8. Prospective calculation of the study size.

#### Publication bias

According to Begg’s test and Egger’s test, all the results, including the UA, Cr, eGFR, WBC, Hb, ALT, and AST, indicated low publication bias (all *P* > 0.05, Table 4). According to the funnel plot, there was also no publication bias regarding any of these reported outcomes (Supplementary Figures S1A-G).

**TABLE 4:** Publication bias assessment.

Items	*P* Value via Begg’s	*P* Value via Egger’s
UA	0.778	0.052
Cr	0.142	0.458
eGFR	0.573	0.683
WBC	1.000	0.936
Hb	0.624	0.332
ALT	1.000	0.940
AST	0.117	0.124

UA, uric acid; Cr, creatinine; eGFR, estimated glomerular filtration rate; WBC, white blood cell; Hb, hemoglobin; ALT, aspartate transaminase; AST, alanine aminotransferase.

## Discussion

Given the background of renal function impairment in renal transplant recipients, the ability of metabolism for UA-lowering drugs in the kidney decreases gradually, which draws the attention of clinicians to prescribe and adjust the dosage of UA-lowering drugs. The metabolites of some UA-lowering drugs, such as allopurinol, are excreted from the urine, which might concern clinicians when administering these drugs (Drugs and Lactation Database, 2018). Febuxostat is a xanthine oxidase inhibitor that is metabolized mainly in the liver, and its metabolites are both excreted in the urine and feces (National Library of Medicine, 2006). Hence, febuxostat seems more suitable for renal transplant recipients with hyperuricemia on the basis of these pharmacological properties. A previous study indicated that the serum uric acid concentration decreases from 481.83 ± 143.36 μmol/L to 302.18 ± 150.50 μmol/L after 1 month of febuxostat administration in renal transplant recipients with hyperuricemia (Baek et al., 2018). In another study, febuxostat reduced the serum uric acid concentration from 459.70 ± 19.98 μmol/L to 328.87 ± 62.57 μmol/L in renal transplant recipients with hyperuricemia (Xu and Zeng, 2021). However, there is still a lack of comprehensive analysis. The current meta-analysis revealed that febuxostat could reduce uric acid, with an MD of 129.981 μmol/L, in renal transplant recipients with hyperuricemia. These findings provide relatively comprehensive evidence to demonstrate the UA-lowering effect of febuxostat in renal transplant recipients with hyperuricemia to support its application in clinical practice.

Hyperuricemia might also induce graft loss in renal transplant recipients through several mechanisms, including the release of proinflammatory cytokines and damage to endothelial cell function (Nakagawa et al., 2006). Hence, in addition to the UA-lowering effect of febuxostat, its renoprotective effect has received increased attention in renal transplant recipients with hyperuricemia. Febuxostat can effectively alleviate or even reverse fibrosis in renal tissue by reducing the expression of interleukin-6 and transforming growth factor-β1 and decreasing the infiltration of renal interstitial inflammatory cells and the expression of α-agonist proteins and type I collagen (Lin et al., 2017). Furthermore, febuxostat can reduce reactive oxygen species generation, inflammation, and the oxidative stress response, further protecting renal tubular epithelial cells (Miyazawa et al., 2021; Ibrahim et al., 2020). In previous clinical studies, febuxostat increased the eGFR from 75.55 mL/min/1.73 m^2^ to 85.23 mL/min/1.73 m^2^ after a 6 month treatment (Li et al., 2019). The current meta-analysis indicated that febuxostat could increase the eGFR and reduce the Cr level, even though these findings only indicated a boundary of statistical significance. These findings indicate the renoprotective effect of febuxostat in renal transplant recipients with hyperuricemia, but this effect is limited. Furthermore, more *in vivo* and *in vitro* studies are needed to further explore the mechanism by which febuxostat protects renal function. In addition, we also explored the potential source of heterogeneity for UA, and these findings suggest that study design and country might not be the reason for the heterogeneity in UA outcomes. However, the higher dose of febuxostat was associated with lower UA levels, indicating that febuxostat would reduce the UA level in a dose dependent mannar, which also would be a potential source for the heterogeneity of UA.

Febuxostat was reported to be safe in previous studies. Similarly, our study revealed that febuxostat administration had almost no effect on WBC, Hb, ALT, or AST levels in renal transplant recipients with hyperuricemia, which indicated that febuxostat has a good safety profile in the current study. However, few studies have comprehensively explored the safety of febuxostat in these patients, therefore, the safety of febuxostat should be further explored. In addition, as previous study reported, febuxostat might also induce the cardiovascular and hepatic adverse effects (Gray and Walters-Smith, 2011; Kraev et al., 2023). Besides, the U.S. Food and Drug Administration (FDA) also warns its potential increased risk of cardiovascular mortality (available at: https://www.fda.gov/drugs/drug-safety-and-availability/fda-adds-boxed-warning-increased-risk-death-gout-medicine-uloric-febuxostat). Hence, it should be reconsidered to balance the clinical efficacy benefit and safety risk during its clinical application. Recently, some studies also propose the potential regimen such as the gut microbiota and natural bioactive compounds (Dong et al., 2025; Yang et al., 2022). However, the efficacy and safety of these regimens should be further verified.

It should be noticed that all the studies included in this study are observational studies, and there is still a lack of evidence from RCTs. Compared with RCT, observational studies, especially single-arm studies, are more susceptible to confounding factors and selection bias, and their results may not directly reflect the causal effects of treatment. To evaluate the impact of different research designs on the robustness of the results, we conducted a subgroup analysis, which compared the levels of UA between cohort studies and single-arm studies. The results showed that under the random effects model, there was no statistically significant difference in the UA levels between the two types of studies, suggesting that the overall results were consistent among different study designs. However, under the common effect model, the UA level of cohort studies was lower than that of single-arm studies, suggesting that differences in research design and potential heterogeneity may still have an impact on effect estimation. Therefore, the results obtained from single-arm studies should be interpreted with caution. In the future, it is still necessary to conduct more rigorously designed randomized controlled trials or large-scale prospective cohort studies to further clarify the causal impact of relevant treatments on UA levels.

Several limitations of this study should be noted as follows: (1) Allopurinol was the main treatment before the development of febuxostat; hence, a comparative meta-analysis between febuxostat and allopurinol would be better able to describe the efficacy of febuxostat. Recent META analysis reported that febuxostat demonstrated a better uric-acid-lowering effect than the allopurinol in a broader populations such as population with hyperuricemic patients with or without gout and chronic kidney disease stage 3–5 patients with asymptomatic hyperuricemia (Chen et al., 2025; Fan et al., 2020). However, the limited number on the comparison between the febuxostat and allopurinol in kidney tranplantation made it difficult to carry out this meta-analysis. Hence, further comparative studies between febuxostat and allopurinol are needed. (2) Graft survival is important for assessing the prognosis of renal transplant recipients. However, few studies have reported this outcome, which makes it difficult to analyze this finding. Therefore, further studies are needed to assess the long-term efficacy of febuxostat in renal transplant recipients with hyperuricemia. (3) Even though this meta-analysis retrieved research from many databases, the number of included studies was still small (only ten studies), which was mainly due to the inherent small number of studies in this area. Hence, more studies are still needed to explore the efficacy and safety of febuxostat in renal transplant recipients with hyperuricemia. (4) The grey literature (such as conference abstracts, unpublished studies) was not included in this study, which might introduce the elevated publication bias. (5) Most studies are from China, which might limit the generalizability of our findings to other populations, and further large-scale, multi-ethnic studies are warranted to validate the finding. (6) The immunosuppressive therapy and the detailed HLA data might affect the efficacy of febuxostat in renal transplant recipients with hyperuricemia. However, due to the different regimens in immunosuppressive therapy and lack of data in HLA, these META analyses were hard to perform, and further prospective studies with standardized immunosuppressive protocols and comprehensive immunogenetic data should be carried out. (7) The data about the graft survival, acute rejection and patient mortality were scarce in the current studies, which could be evaluated in the further study.

In conclusion, febuxostat has a good UA-lowering effect in renal transplant recipients with hyperuricemia, while its effect on renal function is uncertain. Given the limited existing evidence, which mainly comes from observational studies, febuxostat is not yet suitable as a routine first-line regimen for hyperuricemia in kidney transplant recipients. It has shown certain therapeutic effects in reducing blood uric acid, but full attention should be paid to safety issues, especially cardiovascular risks. More high-quality research is still needed in the future to verify this.

## Data Availability

The raw data supporting the conclusions of this article will be made available by the authors, without undue reservation.
