# Supplementary material for: Febuxostat effectively reduces uric acid but has a limited renoprotective effect on renal transplant recipients with hyperuricemia: a meta-analysis
Source: Front Pharmacol. 2026 Feb 25;17:1728485. doi: 10.3389/fphar.2026.1728485 (PMC12993176; doi:10.3389/fphar.2026.1728485)
Supplement: Supplementary file 1 [file Table1.docx]

**Supplementary Table 1*.*** Search strategies for PubMed and Cochrane.

| Databases | Search manager or query | Results |
| --- | --- | --- |
| PubMed | (((Febuxostat) OR (uloric) OR (TEI-6720)) AND ((Hyperuricemia) OR (HUA)) AND ((Kidney transplant) OR (Renal transplant) OR (kidney transplantation) OR (renal transplantation) OR (kidney grafting) OR (renal grafting))) | 30 |
| Cochrane Library | #1: Febuxostat  #2: uloric  #3: TEI-6720  #4: Renal Transplant  #5: kidney transplant  #6: Renal Transplantation  #7: kidney transplantation  #8: Renal grafting  #9: kidney grafting  #10: Hyperuricemia  #11: HUA  #12: #1 OR #2 OR #3  #13: #4 OR #5 OR #6 OR #7 OR #8 OR #9  #14: #10 OR #11  #15: #12 AND #13 AND #14 | 10 |
