# Supplementary figures and images for: Febuxostat effectively reduces uric acid but has a limited renoprotective effect on renal transplant recipients with hyperuricemia: a meta-analysis
Source: Front Pharmacol. 2026 Feb 25;17:1728485. doi: 10.3389/fphar.2026.1728485 (PMC12993176; doi:10.3389/fphar.2026.1728485)

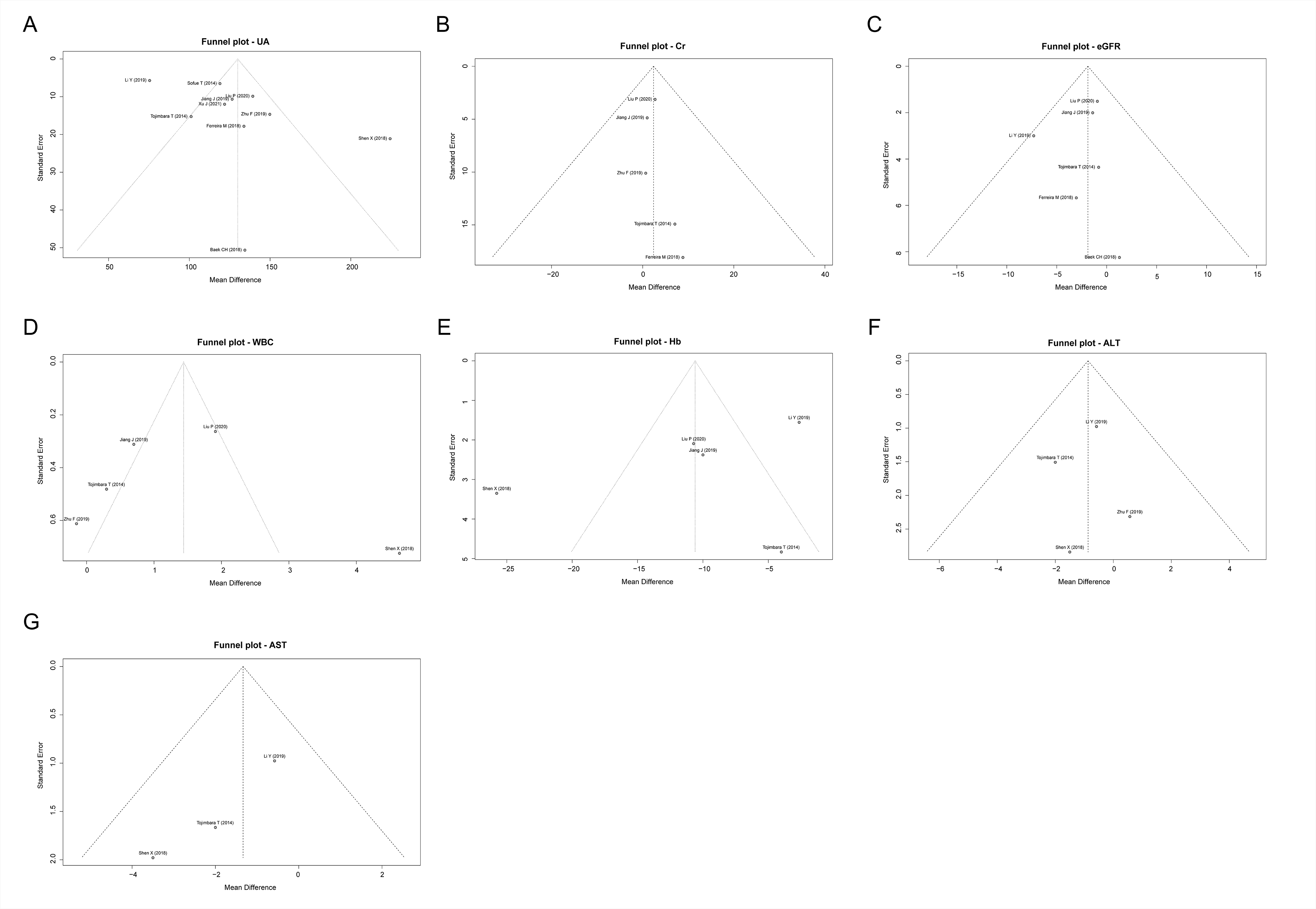

Supplement: Supplementary file 2 [file Image1.tif]
