# Supplementary material for: Febuxostat effectively reduces uric acid but has a limited renoprotective effect on renal transplant recipients with hyperuricemia: a meta-analysis
Source: Front Pharmacol. 2026 Feb 25;17:1728485. doi: 10.3389/fphar.2026.1728485 (PMC12993176; doi:10.3389/fphar.2026.1728485)
Supplement: Supplementary file 3 [file Table2.docx]

**Supplementary Table 2*.*** Additional information on the included studies.

| Study ID | Study type | Dosage of febuxostat stratification (mg/d) | Immunosuppressant therapy | HLA mismatches | Mean baseline UA level (μmol/L) | Achieved UA | | Follow-up duration (months) |
| --- | --- | --- | --- | --- | --- | --- | --- | --- |
|  |  |  |  |  |  | Definition | Rate |  |
| Sofue T (2014) [17] | Cohort | ≤40 | Tac-based: 12 cases;  MMF-based: 13 cases | NR | 499.63 | ≤356.88 μmol/L | ≈50.00% | 12 |
| Tojimbara T (2014) [18] | Cohort | ≤40 | CsA: 15 cases;  Tac: 7 cases | HLA-AB: 2.05±0.89;  HLA-DR: 1.00±0.73 | 475.84 | ≤356.88 μmol/L | 73.00% | ≥7 |
| Baek CH (2018) [19] | Cohort | >40 | Tac: 7 cases;  CsA: 5 cases;  Sirolimus: 1 case;  Mycophenolate: 9 cases | ≥3: 8 cases | 458.59 | ≤356.88 μmol/L | 84.60% | NR |
| Ferreira M (2018) [20] | Single-arm | >40 | Tac: 11 cases;  CsA: 2 cases;  Everolimus: 1 case | NR | 502.61 | ≤356.88 μmol/L | 26.60% | NR |
| Shen X (2018) [14] | Cohort | >40 | Tac-based: 46 cases;  CsA-based: 2 cases | NR | 572.23 | Target level | 73.53% | 6 |
| Jiang J (2019) [21] | Single-arm | ≤40 | Calcineurin inhibitors+anti-proliferative drugs+ glucocorticoids | NR | 501.21 | Male: <420 μmol/L; Female: <360 μmol/L | 66.10% | 12 |
| Li Y (2019) [13] | Cohort | ≤40 | Tac: 9 cases;  MMF: 11 cases;  Prednisolone: 10 cases | NR | 470.82 | Male: <420 μmol/L; Female: <360 μmol/L | 81.80% | 6 |
| Zhu F (2019) [22] | Single-arm | >40 | CsA: 25 cases;  Tac: 79 cases | NR | 497.88 | ≤360 μmol/L | 100.00% | 3 |
| Liu P (2020) [23] | Single-arm | ≤40 | Methylprednisolone+MMF+Mizoribine;  Serum Cr <300 μmol/L: Tac | At least a one-HLA match between the donor and recipient | 521.71 | Male: <420 μmol/L; Female: <360 μmol/L | 71.95% | 6 |
| Xu J (2021)[24] | Cohort | >40 | NR | NR | 450.70 | NR | NR | 3 |

UA, uric acid; NR, not reported; Tac, tacrolimus; IS, immunosuppressant; MMF, mycophenolate mofetil; HLA, human leukocyte antigen; CsA, cyclosporine.
