# Supplementary material for: Febuxostat effectively reduces uric acid but has a limited renoprotective effect on renal transplant recipients with hyperuricemia: a meta-analysis
Source: Front Pharmacol. 2026 Feb 25;17:1728485. doi: 10.3389/fphar.2026.1728485 (PMC12993176; doi:10.3389/fphar.2026.1728485)
Supplement: Supplementary file 4 [file Table5.docx]

**Supplementary Table 5.** Subgroup analyses by dose of febuxostat and follow-up duration on UA.

| Variate/subgroups | Included studies | Pooled estimate [95% CI] | Heterogeneity | Test for overall |
| --- | --- | --- | --- | --- |
| **Dose of febuxostat** |  |  |  |  |
| <40 mg/d | 5 | 111.900 [89.035; 134.766] | I^2^ = 91.606%;  *P* < 0.001 | Z = 9.59;  *P* < 0.001 |
| ≥40 mg/d | 5 | 153.232 [115.328; 191.136] | I^2^ = 78.246%;  *P* = 0.001 | Z = 7.92;  *P* < 0.001 |
| Test for subgroup difference |  | 0.067 | | |
| **Follow-up duration** |  |  |  |  |
| >6 months | 3 | 118.687 [108.450; 128.925] | I^2^ = 0.000%;  *P* = 0.394 | Z = 22.72;  *P* < 0.001 |
| ≤6 months | 5 | 139.842 [93.859; 185.825] | I^2^ = 95.132%;  *P* < 0.001 | Z = 5.96;  *P* < 0.001 |
| NR | 2 | 133.895 [100.938; 166.853] | I^2^ = 0.000%;  *P* = 0.991 | Z = 7.96;  *P* < 0.001 |
| Test for subgroup difference |  | *P* = 0.490 | | |

UA, uric acid; CI, confidence interval; NR, not reported.
