# Supplementary material for: Febuxostat effectively reduces uric acid but has a limited renoprotective effect on renal transplant recipients with hyperuricemia: a meta-analysis
Source: Front Pharmacol. 2026 Feb 25;17:1728485. doi: 10.3389/fphar.2026.1728485 (PMC12993176; doi:10.3389/fphar.2026.1728485)
Supplement: Supplementary file 5 [file Table3.docx]

**Supplementary Table 3.** Summary of the indices before and after-treatment.

| Items | Median (range) | | Mean±SD | |
| --- | --- | --- | --- | --- |
|  | Before | After | Before | After |
| UA (μmol/L) | 498.76 (450.70-572.23) | 371.74 (324.17-395.41) | 495.12±35.03 | 362.569±23.97 |
| Cr (μmol/L) | 127.70 (115.01-160.00) | 126.70 (114.34-151.16) | 134.17±18.69 | 130.11±15.21 |
| eGFR (mL/min/1.73m^2^) | 53.76 (39.50-76.73) | 55.19 (40.30-84.03) | 54.80±12.12 | 56.83±14.66 |
| WBC (10^9/L) | 8.00 (6.16-10.57) | 6.36 (5.86-7.43) | 8.06±1.63 | 6.58±0.75 |
| Hb (g/L) | 121.00 (97.19-123.64) | 125.00 (122.82-134.35) | 116.72±10.99 | 127.35±5.30 |
| ALT (U/L) | 15.89 (13.00-23.64) | 16.36 (15.00-24.22) | 17.11±4.70 | 17.98±4.22 |
| AST (U/L) | 18.00 (17.5-23.64) | 21.00 (20.00-24.22) | 19.71±3.41 | 21.74±2.21 |

SD, standard deviation; UA, uric acid; Cr, creatinine; eGFR, estimated glomerular filtration rate; WBC, white blood cell; Hb, hemoglobin; ALT, aspartate transaminase; AST, alanine aminotransferase.
