# Supplementary material for: Febuxostat effectively reduces uric acid but has a limited renoprotective effect on renal transplant recipients with hyperuricemia: a meta-analysis
Source: Front Pharmacol. 2026 Feb 25;17:1728485. doi: 10.3389/fphar.2026.1728485 (PMC12993176; doi:10.3389/fphar.2026.1728485)
Supplement: Supplementary file 6 [file Table4.docx]

**Supplementary Table 4.** Comparing UA between cohort and single-arm studies.

| Items | Overall studies | Cohort studies | Single-arm studies |
| --- | --- | --- | --- |
| Included studies | 10 | 6 | 4 |
| Heterogeneity |  |  |  |
| I^2^ | 89.955% | 92.332% | 0.000% |
| *P* value | <0.001 | <0.001 | 0.614 |
| Test for overall |  |  |  |
| Z | 10.82 | 5.94 | 22.33 |
| *P* value | <0.001 | <0.001 | <0.001 |
| Random effect model | 129.981 [106.435; 153.526] | 126.627 [84.830; 168.424] | 136.278 [124.317; 148.238] |
| Common effect model | 111.718 [105.360; 118.076] | 102.044 [94.537; 109.550] | 136.278 [124.317; 148.238] |
| Test for subgroup difference |  |  |  |
| Random effect | (-) | *P* = 0.664 | |
| Common effect | (-) | *P* <0.001 | |

UA, uric acid.
