# Supplementary material for: Febuxostat effectively reduces uric acid but has a limited renoprotective effect on renal transplant recipients with hyperuricemia: a meta-analysis
Source: Front Pharmacol. 2026 Feb 25;17:1728485. doi: 10.3389/fphar.2026.1728485 (PMC12993176; doi:10.3389/fphar.2026.1728485)
Supplement: Supplementary file 7 [file Table6.docx]

**Supplementary Table 6.** Univariable meta-regression analyses.

| Items | β [95% CI] | *P* value | I^2^ | R^2^ |
| --- | --- | --- | --- | --- |
| **UA** |  |  |  |  |
| Study type (cohort vs. single-arm) | 10.275 [-40.881; 61.430] | 0.694 | 95.690% | 0.000% |
| Country (China vs. Non-China) | -17.370 [-68.937; 34.198] | 0.509 | 95.060% | 0.000% |
| Dose of febuxostat (per stage) | -14.824 [-27.217; -2.430] | 0.019 | 92.600% | 39.810% |
| Treatment duration (per month) | -0.91 [-29.50; 27.68] | 0.951 | 96.3% | 0.000% |
| **WBC** |  |  |  |  |
| Study type (cohort vs. single-arm) | -1.580 [-5.053; 1.893] | 0.373 | 97.720% | 0.000% |
| Country (China vs. Non-China) | -1.451 [-5.901; 2.998] | 0.523 | 97.920% | 0.000% |
| Dose of febuxostat (per stage) | -0.514 [-1.643; 0.615] | 0.372 | 97.680% | 0.000% |
| Baseline UV level (per μmol/L) | -0.049 [-0.064; -0.031] | <0.001 | 71.120% | 93.880% |
| **Hb** |  |  |  |  |
| Study type (cohort vs. single-arm) | 0.456 [-18.331; 19.243] | 0.962 | 96.67% | 0.000% |
| Country (China vs. Non-China) | 8.136 [-13.664; 29.937] | 0.465 | 97.30% | 0.000% |
| Dose of febuxostat (per stage) | 5.316 [2.254; 8.377] | <0.001 | 84.33% | 77.380% |
| Baseline UV level (per μmol/L) | 0.213 [0.156; 0.270] | <0.001 | 31.74% | 98.030% |

CI, confidence interval; UA, uric acid; WBC, white blood cell; Hb, hemoglobin. The “Dose of febuxostat” was treated as a continuous variable based on the original records from the included studies, with values assigned as follows: 10-20 mg/d = 1, 20 mg/d = 2, 10-40 mg/d = 3, 20-40 mg/d = 4, 40 mg/d = 5, 40-80 mg/d = 6.
